# Review on CO_2_ Capture Using Amine-Functionalized Materials

**DOI:** 10.1021/acsomega.2c03385

**Published:** 2022-10-28

**Authors:** Jannis Hack, Nobutaka Maeda, Daniel M. Meier

**Affiliations:** Institute of Materials and Process Engineering (IMPE), School of Engineering (SoE), Zurich University of Applied Sciences (ZHAW), Winterthur CH-8400, Switzerland

## Abstract

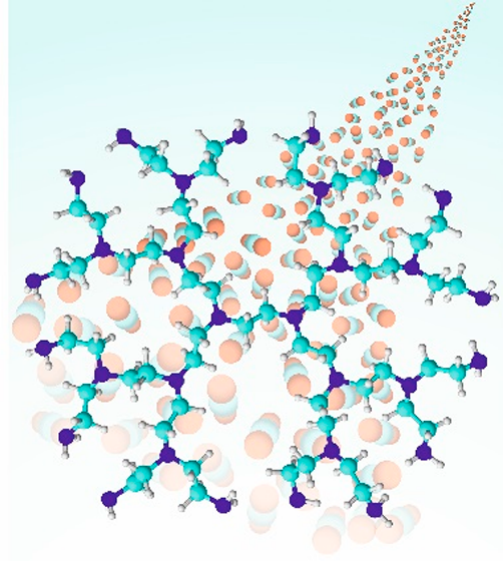

CO_2_ capture from industry sectors or directly from the atmosphere is drawing much attention on a global scale because of the drastic changes in the climate and ecosystem which pose a potential threat to human health and life on Earth. In the past decades, CO_2_ capture technology relied on classical liquid amine scrubbing. Due to its high energy consumption and corrosive property, CO_2_ capture using solid materials has recently come under the spotlight. A variety of porous solid materials were reported such as zeolites and metal–organic frameworks. However, amine-functionalized porous materials outperform all others in terms of CO_2_ adsorption capacity and regeneration efficiency. This review provides a brief overview of CO_2_ capture by various amines and mechanistic aspects for newcomers entering into this field. This review also covers a state-of-the-art regeneration method, visible/UV light-triggered CO_2_ desorption at room temperature. In the last section, the current issues and future perspectives are summarized.

## 1 Introduction

Since the onset of the Industrial Revolution in the 18th century, the atmospheric concentration of carbon dioxide increased from ca. 280 ppm^1^ to 417 ppm in 2022. The climate change and sea level rise of 1–3 mm per year arise from the record-high level of atmospheric CO_2_ concentration. Besides, CO_2_-induced ocean acidification is considered to have an impact on organisms and the ecosystem.^2,3^ Provided that no action is taken, the atmospheric CO_2_ concentration is estimated to rise up to 450 ppm by 2050.^4^ Because of surging public concerns about potential environmental damage, carbon capture and storage/sequestration (CCS)^5^ or utilization (CCU)^6^ became one of the key research topics in the 21st century. CCS aims at geological storage of CO_2_ in the deep underground. On the other hand, CCU is a concept in which captured CO_2_ is utilized as a carbon source for chemical feedstocks such as fuels and fine chemicals. The scientific community and industry are gradually coming to a conclusion that CCS has limitations, taking into account the economic feasibility. Hence, industries are shifting toward exploration of CCU technologies.^7^ In any case, CO_2_ capture is the very first step and thus the most critical technology to pave the way for facilitating the industrialization of CCS and CCU. In the industrial sector, pressure swing adsorption (PSA) and amine scrubbing have traditionally been applied for some decades.^8^ PSA, which has been the main technology in the last a few decades, separates hydrogen out of other gases, including CO_2_. Since PSA aims at purification of H_2_, the resulting concentration of CO_2_ collected from off-gas is too low to utilize CO_2_ for further chemical processes.^8^ On the other hand, amine scrubbing has been applied since 1930 and allows CO_2_ collection with high purity (>99%).^9^ CO_2_ is captured by using monoethanolamine solution (20–30 wt % in water) and then released at 100–120 °C. Amine scrubbing produces a huge amount of degraded solvent as waste. Therefore, the current scrubbing technologies are energy intensive and therefore not very economical. To improve CO_2_ capture technology, two major drawbacks of amine scrubbing need to be overcome: (1) energy-inefficient regeneration at high temperature and (2) waste management of the degraded solvent containing amines. In light of this, CO_2_ capture using solid materials has recently come under the spotlight.^10^ CO_2_ adsorption on solid materials occurs at solid–gas interfaces and thus requires no solvent, leading to the complete elimination of the solvent disposal process. Normally, CO_2_ adsorption on solid materials is carried out at ambient temperature, followed by regeneration of the materials at 80–120 °C to release CO_2_. Therefore, high energy input during the regeneration process is still inevitable. Solid adsorbent-based technology can be applied for two different capturing concepts. One is the direct air capture (DAC) which captures CO_2_ directly from the atmosphere. The other is CO_2_ capture from the off-gas of industrial sectors such as power plants and factories for fine chemical synthesis. DAC is considered to be a “negative emission technology” because already released CO_2_ in the air (417 ppm) has to be captured. However, DAC is much less cost-effective compared to CO_2_ capture from exhaust streams because of the low concentration of CO_2_ in the air and thus the associated thermodynamic barrier.^4^ On the other hand, CO_2_ capture from industrial off-gas is considered as “zero emission technology” in order not to increase the current CO_2_ level any further. Depending on emission sources, the CO_2_ concentration differs in a wide range of 3–100%.^11^ For example, the off-gas from coal-fired power plants contains 10–15% CO_2_, whereas fermentation plants for ethanol production emit 98–99% CO_2_.^11^ Hence, CO_2_ capture from these plants is thermodynamically favored and can be cost-effective. Public concerns about global warming and awareness of the importance of CO_2_ removal make both “negative emission technology” and “zero emission technology” a high priority. Such an urgent demand has lately been extending the horizon of solid adsorbent materials with new concepts, and in the past decade we have witnessed tremendous advance in this field.^10^ Porous materials have been extensively explored, targeting high CO_2_ adsorption capacity such as carbonaceous adsorbents, ionic liquids, zeolites, metal oxides, and metal–organic frameworks (MOFs). The overview of different adsorbents is summarized in a previously published review article.^10^

In this review, we outline properties, advantages, disadvantages, and future perspectives of amine-functionalized materials, foreseeing their potential for high CO_2_ adsorption capacity, relatively low regeneration temperature, cost-effectiveness, and industrial applications. This article not only summarizes different amine-based materials but also sums up the effects of impurities in off-gas streams and adsorption mechanisms and process engineering.

## 2 Amine-Functionalized Solid

Amine-functionalized solid adsorbents light a path for mitigating the issues encountered by liquid amine scrubbing, such as the loss of volatile amines by vaporization. The support materials with high surface area and porosity are functionalized with amines, such that the amines are well-dispersed as uniformly as possible. Hence, the functionalized materials possess the positive properties of both components (an amine and a support). The support materials are often functionalized in two different ways: wet impregnation or anchoring on the surface with covalent bonds. This chapter discusses what types of amines and solid support materials have already been explored. Table 1 lists adsorbent materials reported by different research groups in the last 15 years.

**Table 1:** Summary of CO_2_ Adsorption Performance of Different Adsorbents

Support	Absorbent	Amine loading [wt %]	Adsorption capacity [mmol/g]	Adsorption temperature [°C]	Desorption temperature [°C]	Adsorption conditions	Reference
SBA-15	functionalized by different aminosilanes	-	2.2 to 3.2	25	95 to 105	pure CO_2_, static	Boukoussa et al.^31^
SBA-15 and fumed silica	TEPA, impregnated	-	3.25 to 3.97	75	115	15% CO_2_, no information about gas flow	Chao et al.^32^
β-zeolite	TEPA, impregnated	38.4	2.90	30	135	10% CO_2_, 30 mL/min	Fischer et al.^12^
protonated titanate nanotubes (PTNTs)	TEPA, impregnated	60	4.13	75	100	10% CO_2_, 10 mL/min	Guo et al.^13^
nanoporous titanium oxyhydrate	TEPA, impregnated	60	3.1	60	100	1% CO_2_ and 1% H_2_O, 300 mL/min	Irani et al.^33^
carbon nanotubes (CNTs)	TEPA, impregnated	75	5.0	60	90	10% CO_2_ and 1% H_2_O, 300 mL/min	Irani et al.^14^
PMMA (HP2MG)	TEPA and modified TEPA, impregnated	-	2.58 to 4.30	25	100	pure CO_2_, 200 mL/min	Jo at al.^15^
silica gel	TEPA and PEI, impregnated	40	2.64 to 3.32	75	100	pure CO_2_, 100 mL/min	Jung at al.^34^
PAN carbon fibers	TEPA and TETA, impregnated	50	4.30 to 5.44	25	90	10% CO_2_, 30 mL/min	Kuang at al.^35^
mesoporous SiO_2_ (MCM-41)	EPA, DETA, TEPA, and PEHA, impregnated	40	1.19 to 2.34	35	100	10% CO_2_, no information about gas flow	Liu at al.^17^
TiO_2_	monoethanolamine, impregnated	40	1.09	45	90	1% CO_2_, 300 mL/min	Sun at al.^36^
MR10 (LDPE)	BPEI, impregnated	50	2.64	25	70	different concentrations of CO_2_, 3 mL/min	Wang at al.^22^
fumed silica	BPEI and IL, impregnated	30 and 10	-	25	50	5000 ppm of CO_2_, 200 mL/min	Weisshar at al.^23^
nanosilica	LPEI, impregnated	different loadings	1.0 to 3.5	different temperatures	50–60	different concentrations of CO_2_, 3 mL/min	Zhang at al.^21^
-	IRMOF-74-III-(CH_2_NH_2_)_2_, alkylamine functionality	-	1.2	25	120	pure CO_2_, static	Flaig et al.^29^
-	N-containing polymer NUT-4, imine linker	-	6.9	25	25	10% CO_2_	Geng et al.^27^
-	N-doped copolymer		3.1	0	-	pure CO_2_, static	Qi et al.

### 2.1 Type of Amines

**Figure 1:**
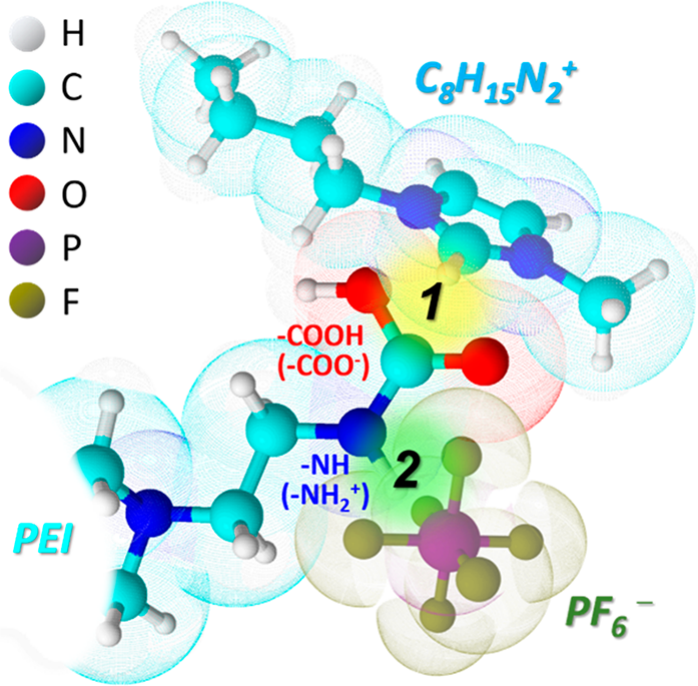
Proposed interaction of BPEI and ionic liquid. Copyright 2016 American Chemical Society. Reprinted with permission from ref (23).

As described in the Introduction, the monoethanolamine solution has been used as a main sorption component for amine scrubbing. Since monoethanolamine is not suitable for the functionalization of solids due to its high volatility and the small adsorption capacity of CO_2_, longer-chain amines have often been employed in the past. A prime example in this respect is tetraethylenepentamine (TEPA), which contains five amine groups and thus has the theoretical capacity of chemisorption of up to five CO_2_ molecules per one TEPA molecule. Because the past research mainly focused on the adsorption capacity, the high adsorption capacity of TEPA attracted much attention.^12−16^ As a result, TEPA has become one of the most widely used amines for the functionalization of solid materials. Besides, other ethyleneamines, such as triethylenetetramine (TETA) and pentaethylenehexamine (PEHA), are also suitable for the functionalization as they have similar performance in CO_2_ adsorption.^17^ Recently, the amine-containing polymer, i.e., polyethylenimine (PEI), has also come to the forefront of research. PEI has two different structures: branched polyethylenimine (BPEI) and linear polyethylenimine (LPEI).^18−21^ Due to the significantly lower price and availability in the market, BPEI was studied more extensively compared to LPEI. Both PEI types have adsorption capacity similar to or slightly lower than TEPA. However, the regeneration temperature of PEI is much lower, especially LPEI, compared to shorter ethyleneamines.^21,22^ Another exciting aspect of the use of PEI is the influence of the polymer size. Smaller PEI molecules are better suited for the impregnation of porous materials because they can diffuse more uniformly into the pores than large PEI molecules. Larger PEI molecules, on the other hand, are less soluble in water, resulting in better regeneration stability under humid conditions. This is an important factor considering long-term operations. As shown in Table 1, different adsorption temperatures 25–75 °C were chosen for each of the reports. The concept of such adsorption conditions was built upon the exploitation of the off-gas heat from industrial sectors. The source of CO_2_ emission from the industrial sectors is normally higher than room temperature, e.g., coal and petroleum power plants (40–65 °C).^11^ Therefore, the adsorption performance in this temperature range is important for industrial applications. Due to the climate change and global concerns about energy and the environment, the research direction is shifting toward how to lower the regeneration temperature without compromising the high adsorption capacity. Weisshar et al. reported that a hybrid adsorbent of BPEI with ionic liquid released CO_2_ even at 50 °C.^23^ The zwitterions formed by the reaction of amines and CO_2_ interact with counterion pairs of the ionic liquid, leading to the weakening of the C–N bond as shown in Figure 1.

Yogo et al. reported a different approach to improving both adsorption and desorption properties by chemical medication of amino groups.^24^ TEPA substituted with bulky alkyl groups was found to enhance the adsorption capacity and to lower the regeneration temperature. They achieved 88% efficiency in the desorption at 60 °C by sweeping an inert gas.

### 2.2 Support Materials

The choice of an appropriate support material is driven by several factors. Two important properties for the support material are the thermal stability and chemical inertness. Accordingly, the support material should not be decomposed at relatively high temperature and should not react with amines during functionalization so that the amines remain available for the chemisorption of CO_2_. Table 1 shows the various support materials tested in recent years. It is worth noticing that all supports reported fulfill the two criteria mentioned above. However, additional properties must be considered to finely tune the adsorption and desorption performance. For example, additional decisive properties are specific surface area, porosity, and pore volume, whereby the adsorption performance is interrelated. The specific surface area plays an important role in impregnating amines onto the support material uniformly. Support materials with high surface area can impregnate a larger amount of amines, leading to higher adsorption capacity. Because BPEI is a highly viscous liquid, thick liquid BPEI layers are formed on support materials with low specific surface area. The thick liquid layers inhibit gas diffusion, resulting in the low efficiency for CO_2_ adsorption. Furthermore, the porosity and the pore volume are important for the diffusion of gaseous molecules through the support material. Materials with high porosity and large pore volume are favored due to better mass transport through the support material. Such a property can enhance the adsorption capacity as well as rapid desorption of CO_2_. Thus, the choice of support material drastically influences the performance of the adsorbents.^25^ Furthermore, the number of acid sites on the surface also has an influence on the sorption properties of the amine. The beneficial effects of acidic sites of the support material can be attributed to the interaction between the acid sites and the amine groups. The presence of strong surface acidity contributes to the formation of protonated amine species on which CO_2_ weakly adsorbs. This is beneficial to the repeated cycles of adsorption and desorption.^26^ On one hand, the higher thermostability of the entire system and better distribution of the amine on the surface of the support material result in more efficient adsorption.^26^

### 2.3 Functionalized Polymeric Adsorbents

There has been a new strategy to add the CO_2_-capturing property to polymeric compounds such as porous polymers^27,28^ and metal–organic frameworks (MOFs)^29,30^ with amine-functionalized linkers. MOFs are classified as coordination polymers with a metal cation center and organic linker. Yagi and co-workers reported that IRMOF-74-III was functionalized with primary or secondary amines.^30^ IRMOF-74-III-CH_3_ without amine functionalization also adsorbed CO_2_, but the adsorption capacity dropped by 80% under humid conditions. This behavior can be explained by the CO_2_ adsorption to the open Mn sites where H_2_O competitively coordinates. On the other hand, the moisture did not affect the performance of IRMOF-74-III-CH_2_NH_2_, indicating that the CO_2_ uptake occurs with the amine linkers and that the open Mn sites are not available. The regeneration of IRMOF-74-III-CH_2_NH_2_ required 90 °C to remove CO_2_. They extended the synthetic method toward functionalization by diamine and reported that IRMOF-74-III-(CH_2_NH_2_)_2_ adsorbed 2.33 times more CO_2_ than monoamine-functionalized IRMOF-74-III-CH_2_NH_2_.^29^ The complete regeneration was achieved at 120 °C in a vacuum.

Recently, Sun and co-workers have proposed nitrogen-doped porous carbons (NPCs) for CO_2_ capture.^27,28^ The NPCs were prepared through polymerization of 2,4,6-tris(chloromethyl)mesitylene (TCM) and *p*-phenylenediamine (PD) to a polymer of NUT-4 (NUT stands for Nanjing Tech University). The obtained NUT-4 was further carbonized at elevated temperatures (400–700 °C) to obtain NPCs. The CO_2_ adsorption capacity of 6.9 mmol/g was achieved with NPCs containing CO_2_-philic N sites. The complete regeneration of the adsorbent was possible under mild condition (25 °C, 30 mmHg, 60 min).

## 3 Moisture Effect

**Figure 2:**
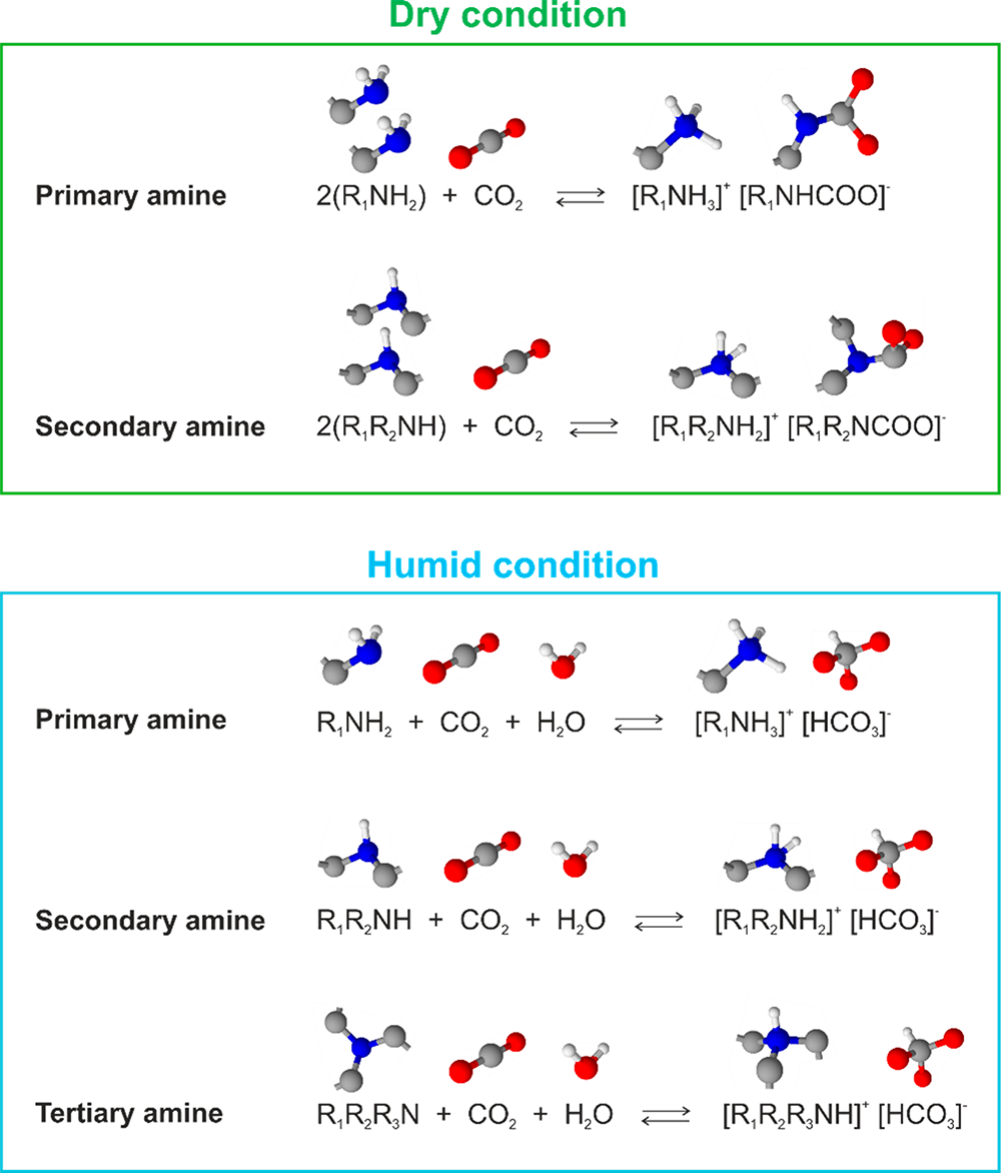
Examples of reaction schemes of CO_2_ adsorption under dry and humid conditions.

Amine-functionalized solid materials are advantageous over porous adsorbents because of their high tolerance against moisture. In most of the cases, the presence of moisture even enhances the CO_2_ adsorption capacity^14,20,22,33,36−40^ yet keeps the regeneration temperature in the same range.^23^ The atmosphere contains 18–60% humidity on average over the year. The exhaust gas from power plants also contains moisture. Therefore, both DAC and CO_2_ capture from industrial off-gas using amine-functionalized materials can greatly benefit from the enhancement effect of CO_2_ adsorption by moisture. Figure 2 shows typical examples of reaction schemes of amines with CO_2_ under dry and humid conditions.^14^ Under dry conditions, two primary amines react with one CO_2_ molecule to form an ammonium ion and carbamate ion. The secondary amines follow the same reaction path. Therefore, the maximum efficiency can only be 0.5 mol of CO_2_ per mole of amine. Under humid conditions, both primary and secondary amines react with CO_2_ and H_2_O to form ammonium ions and bicarbonate. Hence, the theoretical value of moles of CO_2_ per mole of primary and secondary amines doubles to 1.0 under humid conditions. Likewise, under humid conditions, CO_2_ and water react with tertiary amines by forming an ammonium ion and bicarbonate and by binding one mole of CO_2_ per mole of tertiary amine. These reactions schemes are examples out of all the potential reaction paths, e.g., the formation of carbamic acid. The actual reaction appears to be more complicated than the schemes described in Figure 2.

**Figure 3:**
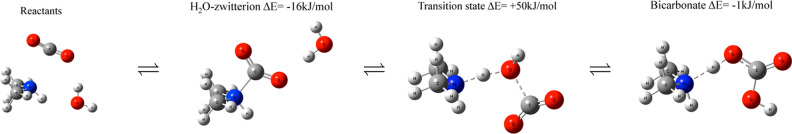
Water-assisted formation of bicarbonate via a zwitterion. Copyright 2016 American Chemical Society. Reprinted with permission from ref (37).

According to the theoretical estimation, the presence of moisture should double the CO_2_ adsorption capacity for primary and secondary amines. Thus, the enhancement effect of moisture depends on the type of amines used and relative humidity (RH). On PEI supported on resin^22^ or polymer,^39^ CO_2_ adsorption capacity increases proportionally with RH. With 100 RH % at a dew point of 25 °C, the CO_2_ breakthrough period was twice as long as that of dry conditions.^23^ The recyclability test was also conducted using a triamine-grafted mesoporous material.^38^ Under dry conditions, CO_2_ uptake dropped by 15% after 700 cycles because of the formation of a stable urea, whose reaction is not reversible. Under humid conditions, the same CO_2_ adsorption capacity was kept over 700 cycles. H_2_O was reported to react with urea to form carbamate, leading to the long-term durability of the adsorbent. Another striking feature of the moisture effect is that the desorption temperature was never influenced by the presence of moisture even though CO_2_ uptake was doubled.^23^ Normally, the desorption temperature increases for samples with higher loading amount of amine.^41^ A clear answer to this unique effect of the moisture was given by quantum chemical calculations.^37^ As in Figure 3, a H_2_O-stabilized zwitterion is formed during the CO_2_ adsorption process, which helps CO_2_ transport through PEI. Therefore, under humid conditions the diffusion barrier can be minimized.

## 4 Tolerance for Impurities

In this section, the effects of SO_2_, NO_*x*_, CO, and O_2_ on CO_2_-capturing property are summarized. The performance of liquid amine scrubbing with monoethanolamine (MEA) is tremendously deteriorated by the presence of impurities such as SO_2_ and O_2_ in flue gas streams.^42^ Therefore, it is of vital importance to investigate the potential degradation of amine-functionalized materials under such conditions for industrial applications. Xu et al. investigated the effects of impurities in a flue gas on CO_2_-capturing property using 50 wt % of BPEI (Mn = 600) finely dispersed in the pores of MCM-41.^43^ They employed the actual off-gas from a natural gas-fired boiler containing 7.4–7.7% CO_2_, 14.6% H_2_O, ∼4.45% O_2_, 200–300 ppm of CO, 60–70 ppm of NO_*x*_, and 73–74% N_2_. N_2_, O_2_, and CO had little influence on CO_2_ adsorption, while NO_*x*_ slightly decreased the amount of CO_2_ adsorbed. However, CO_2_ was captured 3000 times more than NO_*x*_, demonstrating that BPEI/MCM-41 can efficiently separate CO_2_ from the flue gas. Rezaei et al. reported single-component adsorption^44^ and multicomponent adsorption^45^ using SO_2_, NO_*x*_, and CO_2_ with different amine types. They demonstrated that SO_2_ greatly influenced CO_2_ adsorption at 35 °C but had much less influence at 75 °C.^44^ This phenomenon originates from the fact that SO_2_ adsorption capacity decreases with an increase in temperature, while CO_2_ adsorption capacity increases. The coadsorption experiments showed that the presence of SO_2_ considerably deteriorates CO_2_ adsorption performance.^45^ Among all the amine types, secondary amine was less influenced by SO_2_. Coadsorption with NO has no impact on CO_2_ adsorption, whereas NO_2_ slightly suppressed CO_2_ adsorption capacity.^45^

The degradation of amine-functionalized materials by oxygen was also extensively studied.^46−51^ Normally, the off-gas from power plants and some fine chemical factories contain a certain concentration of oxygen. Therefore, O_2_ tolerance of the amine-based materials should be taken into account for industrial applications with long-term operation. The reason for the degradation by O_2_ has never been clearly demonstrated. Heydari-Gorji et al. carried out infrared spectroscopic and NMR analysis of a triamine-grafted material (TRI).^48,49^ Upon the TRI deactivation, a new IR band emerged at 1665–1680 cm^–1^, but only a minor change was confirmed by ^13^C NMR except for a weak peak at 158 ppm. What these changes indicate remain uncertain. However, they assumed that the possible formation of imine, carbamic acid, and nitrone cannot be ruled out. Further research is required to draw a solid conclusion on the oxidative degradation mechanism of TRI. Bali at al. thoroughly investigated the oxidative degradation of PEI and poly(allylamine) (PAA) on mesoporous alumina support employing infrared, Raman, and ^13^C NMR spectroscopy.^47^ Their data clearly proved the formation of a C=O bond associated with deactivation of PEI. They concluded that undesirable formation of amides, acids, and imides leads to lower basicity of the nitrogen species in the polymer and thus low CO_2_ adsorption capacity. According to their findings, PAA may serve as a better adsorbent with high oxidative stability. Ahmadalinezhad et al. also came to a similar conclusion by using 1D and 2D NMR techniques.^46^ BPEI is the most unstable amine among the amines tested, and PAA and LPEI can be more resistant to the oxidative atmosphere.

## 5 Mechanistic Studies

The advance in spectroscopy, in situ techniques, and quantum mechanical modeling in the past decade promoted mechanistic investigations to unveil the underlying mechanism of CO_2_ adsorption and desorption. Commonly used techniques in this field are infrared spectroscopy,^23,52−60^ nuclear magnetic resonance (NMR),^61−66^ and density functional theory (DFT).^37,67−70^ This chapter briefly summarizes what information can be gained by each technique and major findings related to CO_2_ adsorption mechanisms.

NMR studies were mainly reported by the group of Jones and colleagues,^61−66^ exploiting ^1^H NMR,^66^^13^C NMR,^64,65^^15^N dynamic nuclear polarization (DNP) NMR,^64^ rotational-echo double-resonance ^15^N(^13^C) and ^13^C(^15^N) (REDOR) NMR,^62^ and two-dimensional ^13^C–^1^H heteronuclear correlation (HETCOR) NMR.^61,63^^13^C NMR is a powerful tool to identify reaction pathways and surface species formed, such as carbamic acid, carbamate, bicarbonate, and urea. In particular, its combination with IR spectroscopy offers a firm understanding of molecular structure because the frequency difference between asymmetric and symmetric stretching vibrations of carboxylate ions provides insight into the molecular interaction.^64^ REDOR NMR is useful to gain information about short- and long-range dipolar coupling between isolated pairs of heteronuclei. ^15^N(^13^C) REDOR NMR identified amide-like species such as carbamic acid and carbamate. Together with its counterpart, ^13^C(^15^N) REDOR NMR, REDOR NMR indicated that carbamate species are isolated from each other.^62^ 2D NMR with HETCOR sequence detects the correlation of two different nuclei (c.f., ^13^C and ^1^H) via single-bond spin–spin coupling and thus unveils which proton is bonded to which carbon groups. Chen et al. reported that ^13^C and ^1^H HETCOR NMR detected two distinct bicarbonate species at 100 K, which are coupled to different protons, respectively.^63^ Their follow-up study evidenced that one bicarbonate species is coupled to H_2_O molecules present on the walls of the mesoporous material, while another bicarbonate species is coordinated to H_2_O molecules in the pores.^61^

DFT calculations contributed considerably to understanding what species is favorably formed and what reaction path is reasonable for the reaction of amines with CO_2_.^37,67−70^ Early stage research was reported by Mebane et al. that the formation of a zwitterion was unstable in a polar environment of anhydrous PEI.^67^ However, a dielectric medium under humid conditions stabilizes the zwitterion. They also proposed linear and ring topologies to be responsible for CO_2_ diffusion in the bulk PEI under humid conditions.^37^ DFT calculations in combination with adsorption isotherms demonstrated that CO_2_ uptake caused the volume expansion of the amine layers and thus the decrease in the pore volume.^69^ Therefore, sticky amines with high viscosity might increase the diffusion resistance and have a negative impact on CO_2_ adsorption property.

In situ infrared spectroscopy is the most widely utilized technique to follow the reaction path in this field because the setup is relatively cheap and can be operated easily.^23,52−60,64,71^ Since there is an extensive review on IR spectroscopic studies published in 2019,^54^ we only describe major findings briefly and update that what has been newly reported since then. A number of reports contributed to assign IR bands observed during CO_2_ adsorption such as carbamic acid, carbamate-physisorbed CO_2_, and ammonium ions. The detailed band assignment is given in a previous review article.^54^ The most frequently used amine, TEPA, was also investigated by in situ IR spectroscopy. By increasing the thickness of TEPA layers, the interaction between amines was enhanced, forming zwitterions with NH and NH_2_ groups.^59^ This phenomenon suppresses CO_2_ gas diffusion in a thicker layer of liquid TEPA and leads to high-temperature desorption of CO_2_ at 100 °C. The addition of polyethylene glycol (PEG) with low molecular weight TEPA was reported to increase the ratio of weakly adsorbed CO_2_, whose band emerged at 2627 cm^–1^ (NH_2_–O).^57^ PEI also possesses similar characteristics of IR bands.^56^ IR spectroscopy together with thermogravimetric analysis (TGA) provided important information about BPEI behavior at different temperatures. Due to the high viscosity of liquid BPEI, adsorption capacity increased with temperature because of the reduction of the viscosity.^56^ There are several reports on amine-grafted/-immobilized materials.^52,53,58,60^ The IR spectroscopic features and behavior were similar to that of TEPA and BPEI. Effects of NO_2_ and SO_2_ on CO_2_ adsorption were also investigated by in situ IR spectroscopy.^55^ Those acidic gases form NH–NO_2_ and NH–SO_2_ complexes assigned at 1650 cm^–1^, leading to the deterioration of CO_2_ adsorption performance. NO_2_ has less influence on the amount of CO_2_ adsorbed even at 200 ppm, but the presence of SO_2_ leads to the considerable reduction of CO_2_ adsorption above 50 ppm. Weisshar et al. have recently demonstrated that in situ IR spectroscopy could be a useful tool to monitor the weakening of the C–N bond of carbamic acid and carbamate.^23^ Such an attempt would be beneficial for designing an adsorbent material with low-temperature regeneration.

## 6 Reactor and Process Design

### 6.1 Vacuum Swing Desorption

**Figure 4:**
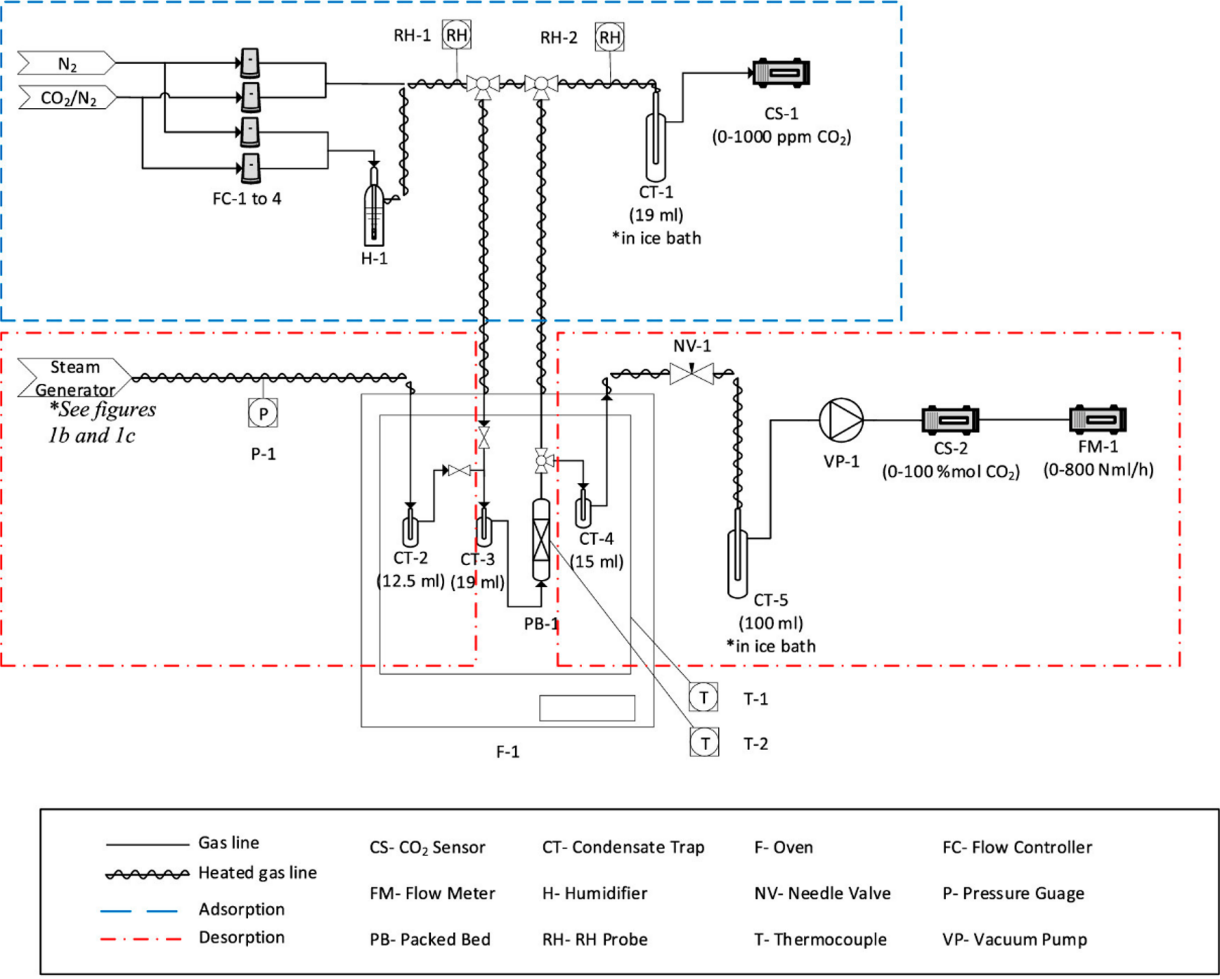
Water-assisted formation of bicarbonate via a zwitterion. Copyright 2016 American Chemical Society. Reprinted with permission from ref (72).

Temperature vacuum temperature swing desorption (TVSD) is considered to be useful because a high-level vacuum is not required.^72^ The process optimization is critical to not waste time, energy, and CO_2_ gas during the desorption process so that the maximum efficiency for collecting pure CO_2_ can be achieved. In the TVSD process, CO_2_ is first chemisorbed on an absorbent material at atmospheric conditions (typically 25 °C and 1 bar). After saturation of the material with CO_2_, the chamber with the adsorbent is closed and evacuated by a vacuum pump, removing the excess gas at the begging and weakly adsorbed CO_2_. In order to desorb further CO_2_, the absorbent bed is heated to the desorption temperature.^73^Figure 4 displays the schematic diagram of a typical TVSD setup. There is also an attempt without any temperature swing, i.e., vacuum swing desorption (VSD), operated at 90 °C.^74^ VSD can achieve 95% CO_2_ purity and 90% recovery. Due to the high-temperature operation, the degradation of CO_2_ adsorption capacity is expected with repeated cycles. Hence, the fabrication of thermally stable adsorbents must be taken into account.^74^ Compared to VSD, TVSD is more efficient when higher CO_2_ concentration is required. In some cases, >99% CO_2_ can be achieved with 0.23 mmol/g/h of the desorption rate.^72^ Under humid conditions during the vacuum process, the desorption rate increased to 3.75 mmol/g/h, confirming the beneficial kinetics assisted by steam. However, additional energy is required for the desorption of water if H_2_O is coadsorbed with CO_2_. The recyclability test revealed that 8% loss in CO_2_ adsorption capacity was observed after 50 cycles (>1500 h).^72^ Gebald et al. also reported cyclic experiments using TVSD with amine-functionalized cellulose.^75^ 100 cycles of adsorption and regeneration at 90 °C caused 2% loss of the N content. However, this report showed a high potential that TVSD can be well applied for DAC.

### 6.2 Steam-Stripping Regeneration

Instead of TVSD, steam-stripping regeneration combined with temperature swing desorption (TSD) has been explored to collect pure CO_2_.^76−82^ In the regeneration process, a stream saturated with H_2_O carries a mixture of CO_2_ and H_2_O, and then a concentrated CO_2_ can be obtained by compression and condensation.^83^ There is a report on studying how to minimize the energy consumption, employing superheated steam from low-pressure steam turbine and a heat exchanger.^76^ This so-called “direct steam-stripping process” was found to lower the energy consumption by 23.2% compered to a conventional stripping method. The steam stripping can also be combined with vacuum swing adsorption, named steam-aided vacuum swing adsorption (SA-VSA).^77^ Yogo et al. demonstrated highly efficient CO_2_ capturing performance using a system with three columns: the first column for adsorption, the second column for rinsing, and the third column for desorption.^77^ The SA-VSA process with an amine-impregnated mesoporous MSU-F silica can achieve >98% of CO_2_ purity and >93% of recovery rate. They also estimated the required heat for the regeneration to be much lower than that of liquid amine scrubbing and other amine-based solid adsorbents.

A challenge for its industrial application is the H_2_O tolerance of amines at elevated temperature. After steam treatment, the adsorption capacity drops to a certain degree.^83,84^ For long-term operations, the fabrication of robust amine-based adsorbents is desired. Sayari et al. reported that amine-grafted SBA-15 showed high hydrothermal stability, and its exposure to steam for 48 h caused no change in the adsorptive and structural properties.^85^ A choice of support materials is also a key factor for the steam tolerance. Upon exposure to steam, alumina support is partially transformed into boehmite, but it does not alter the amine efficiency.^80^ Therefore, γ-Al_2_O_3_ is considered to be a promising support material to impregnate PEI.

### 6.3 Reactor Design

The design of the adsorption–desorption chamber is also a crucial factor to achieve high efficiency with amine-functionalized solid materials. There are two flow-reactor types considered: (1) fixed-bed reactor^86^ and (2) fluidized-bed reactor.^87^ In the fixed-bed reactor. The solid adsorbent is placed such that its position always remains the same when a gas stream flows through the fixed bed.^86^ On the contrary, in the fluidized bed reactor, the adsorbent powders are swirled up with a gas stream and fly around inside the chamber.^87^ The advantage of the fixed-bed reactor is that the process engineering and reactor design are much simpler and cheaper than the fluidized-bed reactor. Furthermore, a significantly larger quantity of adsorbent can be used per unit of space, which results in better process efficiency. However, the fluidized-bed reactor benefits from high adsorption rate and uniform heat distribution because the adsorption process is exothermic.^87^

### 6.4 Process Design and Cost Estimate

A techno-economic analysis (TEA) was considered to assess the applicability of reactor systems with amine-functionalized solids for large-scale CO_2_ capture from exhaust gases. This included four different reactor types (fixed bed, fluidized bed, moving bed, and rapid thermal swing) and was compared with the liquid amine scrubbing often used today. By integrating the adsorption/desorption and heating/cooling processes, TEA estimated a cost of the process by each reactor which falls into the range of 48.1–75.2 $/t-CO_2_ and a heating recovery of 45–58%. These results support the use and need for CO_2_ capture for flue gas purification.^88^ The lowest price of 48.1 $/t-CO_2_ can be achieved with the fixed-bed adsorption configuration. Considering the current cost of 62.8 $/t-CO_2_ with amine scrubbing using 30 wt % of MEA, CO_2_ capture with amine-functionalized solid materials has a high potential to compete in the market.

Zhao et al. designed and evaluated a 200 kW_th_ pilot reactor for the energy balance of three different regeneration processes (thermal regeneration with CO_2_ stream, vacuum regeneration, steam-stripping regeneration).^89^ Amine-functionalized resin was selected to evaluate the efficiency and energy needs for each of the regeneration processes. The least energy is required for the thermal regeneration. Compared to the liquid amine scrubbing often used today, the energy consumption can be reduced by 30% or more. However, if the thermal regeneration is supported by an additional vacuum system, the energy consumption is higher than the thermal process itself but still much lower than the liquid amine scrubbing. In addition, the whole reactor is complicated and more expensive when the vacuum is applied. The steam-stripping regeneration requires the most energy, but the process can be greatly optimized when the steam condensation and heat recovery are taken into account.^89^ The total annual cost also depends on the plant size. Mazzotti et al. performed a techno-economic assessment and found that the cost of commercially available adsorbent considered gas had much less impact on the process cost, but the size and shape of the adsorption system critically affect the investment cost.^90^ The adsorption-based process can be competitive at the small scale (less than 100 tons of flue gas per day) and low recovery rate (less than 40%). However, the classical liquid amine scrubbing is still the most cost-effective at most of the plant sizes and recovery rates.

To perform a cost estimation for CO_2_ capture and concentration (CCC) technology, a model was created to calculate the cost per ton of CO_2_ captured for a 500 MW power plant. The absorbent used was zeolite 13X, which can also be replaced by an amine-functionalized material. Based on the calculations, a tremendous amount of energy can be saved when the concentration of CO_2_ in the flue gas is high enough. Besides, the adsorption efficiency of the reactor can be increased by shortening the reactor length with the larger diameter. At an off-gas CO_2_ concentration of 15%, the cost estimate was $32.8 to $34.4 per ton of CO_2_ captured.^91^

## 7 Light-Triggered CO_2_ Adsorption and Desorption

In this section, a state-of-the-art CO_2_ desorption method is described. In the past decade, photosensitive functional groups are applied for low-temperature regeneration of adsorbents by visible and/or UV light.^92−94^ This method allows CO_2_ adsorption–desorption cycles to be operated under mild conditions (1 bar and room temperature). Lyndon et al. reported that a zinc-based MOF with 4,4′-dicarboxylate (AzDC) and *trans*-1,2-bis(4-pyridyl)ethylene (4,4′-BPE) as a framework released CO_2_ under UV light irradiation at 30–31 °C.^92^ In situ FT-IR spectroscopy detected the change in C–C–C and C–C–N bending modes, originating from *cis*–*trans* isomerization of Zn(AzDC)(4,4′-BPE)_0.5_ under UV irradiation. The desorption capacity was 42% under static irradiation conditions and 64% under dynamic measurements.

Sun and co-workers reported photosensitive MOFs functionalized with azobenzene for CO_2_ adsorption–desorption by UV and visible lights.^93^ Tetraethylenepentamine (TEPA) was impregnated onto an UiO-type MOF functionalized with azobenzene (U-azo). Azobenzene changes its form between *trans* and *cis* under visible and UV lights, respectively. Exploiting this phenomenon, CO_2_ bound to TEPA is released by UV light irradiation. U-azo has the CO_2_ adsorption capacity of 43.4 cm^3^/g (ca. 1.94 mmol/g), among which 45.6% of adsorbed CO_2_ can be released. They also demonstrated that CO_2_ can be selectively separated using this principle out of gas mixtures of CO_2_/N_2_ and CO_2_/CH_4_. They further developed a novel adsorbent which works only with visible light.^94^ MCM-41 was functionalized with (3-aminopropyl)triethoxysilane (APTES) and Disperse Red 1 (DR1). Without irradiation, the photosensitive DR1 is in *trans* configuration, leading to CO_2_ capture on amines. Under visible-light irradiation, DR1 transforms into *cis* configuration, inducing CO_2_ desorption. The CO_2_ adsorption capacity was 32.1 cm^3^/g (ca. 1.43 mmol/g), 40% of which can be reversibly adsorbed and desorbed upon visible-light irradiation.

## 8 Summary

A great deal of research has been reported on CO_2_ capture by amine-based solid materials. In the last two decades, we witnessed a tremendous advance in material synthesis, optimization, and mechanistic aspects. However, practical use in the industrial sector still needs to overcome the following issues:

(1) Long-term thermal stability during the regeneration process
(2) Oxidative degradation
(3) Material fabrication with lower regeneration temperatures
(4) Reactor engineering and process optimization

Especially, point 3, “material fabrication with lower regeneration temperatures”, can mitigate points 1 and 2 because thermal stability and oxygen resistance would be greatly improved when the regeneration is operated in a low-temperature range of 40–60 °C. The cost estimate clearly proved that CO_2_ capture by amine-functionalized solid materials can reasonably be appointed to the position currently taken by the liquid amine scrubbing. The 2021 United Nations Climate Change Conference commonly referred to as the 26th United Nations Climate Change conference (COP26) set new goals for the increase in the global temperature level and CO_2_ emissions. The decision made in COP26 will dramatically change and refine research directions of CCS and CCU technologies. Amine-functionalized solid materials will be one of the main contributing technologies for achieving these goals.
